# Human Cancer Cells Sense Cytosolic Nucleic Acids Through the RIG-I–MAVS Pathway and cGAS–STING Pathway

**DOI:** 10.3389/fcell.2020.606001

**Published:** 2021-01-08

**Authors:** Yuan Qiao, Shan Zhu, Shuanglin Deng, Shan-Shan Zou, Bao Gao, Guoxia Zang, Jing Wu, Yuxue Jiang, Yong-Jun Liu, Jingtao Chen

**Affiliations:** ^1^Institute of Translational Medicine, The First Hospital of Jilin University, Changchun, China; ^2^Department of Neurosurgery, The First Hospital of Jilin University, Changchun, China; ^3^Sanofi Research and Development, Cambridge, MA, United States; ^4^Key Laboratory of Organ Regeneration and Transplantation of the Ministry of Education, The First Hospital of Jilin University, Changchun, China

**Keywords:** cancer cell, nucleic acid, RIG-I, STING, IFN-β

## Abstract

Pattern recognition receptors (PRRs) are germline-encoded host sensors of the innate immune system. Some human cancer cells have been reported to express PRRs. However, nucleic acid sensors in human cancers have not been studied in detail. Therefore, we systematically analyzed the expression, molecular cascade, and functions of TLR3, RIG-I, MDA5, LGP2, cGAS, and STING in human cancer cells. TLR3, TRIF, RIG-I, MDA5, LGP2, and MAVS were expressed in 22 cell lines. The majority of cell lines responded to only RIG-I ligands 5′-ppp-dsRNA, Poly(I:C)-HMW, Poly(I:C)-LMW, and/or Poly(dA:dT), as revealed by IRF3 phosphorylation and IFN-β secretion. IFN-β secretion was inhibited by RIG-I and MAVS knockdown. cGAS and STING were co-expressed in 10 of 22 cell lines, but IFN-β secretion was not induced by STING ligands ISD, HSV60, VACV70, Poly(dG:dC), and 3′3′-cGAMP in cGAS and STING intact cell lines. Further experiments revealed that the cGAS–STING pathway was activated, as revealed by TBK1 and IRF3 phosphorylation and IFN-β and ISG mRNA expression. These results suggest that human epithelial cancer cells respond to cytosolic RNA through the RIG-I–MAVS pathway but only sense cytosolic DNA through the cGAS–STING pathway. These findings are relevant for cancer immunotherapy approaches based on targeting nucleic acid receptors.

## Introduction

Pattern recognition receptors (PRRs) are germline-encoded host sensors of the innate immune system that detect pathogen-associated molecular patterns and self-tissue damage-associated molecular patterns. A major type of PRRs is dedicated to sensing nucleic acids, including DNA and RNA. There are two classes of nucleic acid sensors: those that sense nucleic acids in endosomes, such as TLR3, TLR7, TLR8, and TLR9, and those that sense nucleic acids in the cytosol, such as RIG-I-like receptors (RLRs) and cGAS/STING (Hoffmann and Akira, 2013; Cui et al., 2015; Sparrer and Gack, 2015).

Extensive studies on nucleic acid sensors have focused on their biology and molecular pathways in myeloid cells and antigen-presenting cells (Akira et al., 2006; Gilliet et al., 2008). Upon infection, PRRs recognize nucleic acids and then recruit adaptors and trigger the phosphorylation of IRF3/7, leading to the production of IFN-β and expression of interferon-stimulated genes (Goubau et al., 2013). TLR3, TLR7 and TLR8, and TLR9 recognize dsRNA, ssRNA, and unmethylated CpG DNA, respectively (Kawai and Akira, 2011). Following nucleic acid binding, TLRs undergo conformational changes and recruit adaptor proteins TRIF for TLR3 and MyD88 for TLR7/8/9, leading to IRF3/7 phosphorylation and IFN-β secretion (Akira et al., 2006). RLRs comprise three members: RIG-I (DDX58), MDA5 (IFIH1), and laboratory of genetics and physiology 2 (LGP2; DHX58) (Yoneyama et al., 2015). These RLRs share a DExD/H-box RNA helicase domain and a C-terminal domain, both of which are required for dsRNA detection, while RIG-I and MDA5, but not LGP2, have an N-terminal caspase recruitment domain for interaction with MAVS and downstream signaling (Kawai et al., 2005; Meylan et al., 2005; Seth et al., 2005; Xu et al., 2005; Yoneyama et al., 2005). DNA can also be transcribed into 5′-triphosphate-containing small dsRNA by RNA polymerase III for RLR binding to initiate IFN-β secretion (Ablasser et al., 2009; Chiu et al., 2009). cGAS is a cytosolic DNA sensor that catalyzes the synthesis of cGAMP (Li et al., 2013). cGAMP binds to STING and mediates the activation of TBK1 and IRF3 to initiate IFN-β secretion (Wu et al., 2013). These findings have translated into the development of new adjuvants for the next generation of vaccines and new immunotherapies for cancer that can reverse anti-PD-1/anti-PD-L1 resistance by converting “cold tumors” into “hot tumors” (Fu et al., 2015).

Certain human cancer cells have been reported to express PRRs and respond to cytosolic nucleic acids to produce type I IFNs. TLR3 is expressed in the intestinal epithelium and hepatocytes and senses extracellular Poly(I:C) (Li et al., 2005; Broquet et al., 2011). It is also expressed in human lung epithelial cells to recognize influenza A virus and respiratory syncytial virus (Groskreutz et al., 2006; Le Goffic et al., 2007). Meanwhile, RIG-I and MDA5 are expressed in hepatocytes, intestinal epithelial cells, lung epithelial cells, primary human astrocytes, and glioblastoma, and they respond to cytosolic Poly(I:C) and viruses (Li et al., 2005; Hirata et al., 2007; Wang et al., 2009; Furr et al., 2010; Broquet et al., 2011; Glas et al., 2013; Sugimoto et al., 2014). Upon delivery of Poly(I:C) or viruses into the cytosol, RIG-I or MDA5 expression is significantly increased to initiate the innate immune response. Both cGAS and STING have been found to be expressed in 54.4% and 45.5% of human melanoma and colorectal cancer cell lines, respectively; and cells defective for the cGAS–STING pathway are sensitive to oncolytic DNA virus (Xia et al., 2016a,b). However, there have been no systematic studies on all known nucleic acid sensors in human cancers.

In this study, we systematically analyzed the expression, molecular cascade, and function of the endosomal RNA sensor TLR3; cytosolic RNA sensors RIG-I, MDA5, and LGP2; and cytosolic DNA sensors cGAS and STING in 22 human epithelial cancer cell lines to obtain useful insights into targeting nucleic acid receptors for cancer immunotherapy.

## Materials and Methods

### Cell Culture

All human epithelial cancer cell lines were obtained from the American Type Culture Collection (ATCC). SNB19 and A549 cell lines were maintained in Dulbecco’s modified Eagle’s medium (DMEM)/F12 (Gibco). U251MG, LN-18, U118MG, U87MG, HepG2, Hep3B, HeLa, SiHa, C-33A, MDA-MB-231, MDA-MB-453, Caov-3, SK-OV-3, CFPAC-1, and PANC-1 cell lines were maintained in DMEM (Gibco). NCI-H460, SW480, AGS, MKN45, HCT-8, and THP-1 cell lines were maintained in RPMI-1640 (Gibco). All media contained 10% heat-inactivated fetal bovine serum (FBS) and 1% penicillin–streptomycin. For THP-1 cell differentiation into macrophages, THP-1 cells were treated with 50 nM of PMA for 16 h, after which the cells were cultured for another 48 h without PMA for further experiments. All cell lines were mycoplasma-free and authenticated by short tandem repeat identification by Microread Company (Beijing, China).

### Cell Stimulation

Cells (1.5 × 10^5^) were seeded onto 24-well plates. The next day, the cells were transfected with 5 μg/ml of nucleic acid using 5 μl/ml of Lipofectamine 2,000 (Invitrogen). After 18 h, culture supernatants were harvested to detect IFN-β secretion, and the cells were collected for qPCR and immunoblot analyses at various time points. The nucleic acids used were from InvivoGen [5′-pppRNA (tlrl-3prna), Poly(A:U) (tlrl-pau), Poly(I:C)-HMW (tlrl-pic), Poly(I:C)-LMW (tlrl-picw), ISD (tlrl-isdn), HSV60 (tlrl-hsv60n), VACV70 (tlrl-vav70n), Poly(dG:dC) (tlrl-pgcn), 3′3′-cGAMP (tlrl-nacga), and Poly(dA:dT) (tlrl-patn)].

### ELISA

IFN-β secretion was assessed using an ELISA kit (PBL Interferon Source) according to the manufacturer’s instructions.

### RNA Preparation and Real-Time Quantitative PCR

Total RNA was extracted using the EasyPure RNA kit (TransGen, Beijing, China) according to the manufacturer’s instructions. Reverse transcription was carried out using the EasyScript First-Strand cDNA Synthesis SuperMix (TransGen). The SYBR Green Supermix (Roche) was used for qPCR with the following primers:

GAPDH sense: 5′-GAAGGTGAAGGTCGGAGTC-3′,
GAPDH antisense: 5′-GAAGATGGTGATGGGATTTC-3′;
PKR sense: 5′-AAACCTCTTCGAGGCACAAG-3′,
PKR antisense: 5′-GTTTAGGGCCATCAGCTTCA-3′;
TLR3 sense: 5′-GACAGCCACTCACCTCTTCA-3′,
TLR3 antisense: 5′-AGTGCCTCTTTGCTGCTTTC-3′;
RIG-I sense: 5′-CAGCCGCTTTAGCAGCCA-3′,
RIG-I antisense: 5′-CAAGGAATTGTCTCCCAGTGC-3′;
MDA5 sense: 5′-CTTCTCCTTCCTGATCGTGG-3′,
MDA5 antisense: 5′-TCTCAGCTCCACGCCATT-3′;
LGP2 sense: 5′-GCTGTCATCCTCATTGCTAC TG-3′,
LGP2 antisense: 5′-TGGTGTAGAAATACTCCTTGATG TG-3′;
DHX29 sense: 5′-GAAATTATTCCTGCAAGCCAAT TT-3′,
DHX29 antisense: 5′-TCACCCTTCTTTTTCATGTAG CA-3′;
TRIF sense: 5′-GAAATTATTCCTGCAAGCCAATTT-3′,
TRIF antisense: 5′-TCACCCTTCTTTTTCATGTAG CA-3′;
MAVS sense: 5′-GAAATTATTCCTGCAAGCCAATTT-3′,
MAVS antisense: 5′-TCACCCTTCTTTTTCATGTAG CA-3′;
STING sense: 5′-GAAATTATTCCTGCAAGCCAAT TT-3′,
STING antisense: 5′-TCACCCTTCTTTTTCATGTAG CA-3′;
IFN-β sense: 5′-GAGCTACAACTTGCTTGGATTCC-3′,
IFN-β antisense: 5′-CAAGCCTCCCATTCAATTGC-3′.

*GAPDH* was used as a reference gene to normalize the amounts of cDNA. The relative expression was calculated using the 2^(–ΔΔCt)^ method.

### Western Blot Analysis

Cells were lysed in cell lysis buffer (Cell Signaling, Danvers, MA, United States). Protein concentration was determined by the Bradford assay. Protein (40 μg) was subjected to sodium dodecyl sulfate–polyacrylamide gel electrophoresis (SDS-PAGE) and transferred to polyvinylidene difluoride (PVDF) membranes. Membranes were blocked with 5% bovine serum albumin (BSA) in Tris-buffered saline with 0.1% Tween 20 for 1 h and incubated with the primary antibody overnight. Then, membranes were incubated with appropriate horseradish peroxidase (HRP)-conjugated secondary antibodies and developed using the chemiluminescence system (ECL Advance; Amersham Biosciences). The following primary antibodies were used: anti-Phospho-PKR (3076, 1:1,000), anti-RIG-I (3743, 1:1,000), anti-MDA5 (5321, 1:1,000), anti-LGP2 (12869, 1:1,000), anti-DHX29 (4159, 1:1,000), anti-TRIF (4596, 1:1,000), anti-cGAS (15102, 1:1,000), anti-STING (13647, 1:1,000), and antibodies against phosphorylated (3033, 1:1,000) and whole NF-κB (8242, 1:1,000) from Cell Signaling; anti-PKR (136038, 1:1,000), anti-MAVS (sc-166583, 1:500), and anti-IRF3 (sc-9082, 1:800) from Santa Cruz Biotechnology; and anti-TLR3 (20300418-1, 1:1,000) and anti-β-actin (20312755-1, 1:3,000) from Bioworld. The secondary antibodies were HRP-conjugated goat anti-rabbit (130549, 1:2,500, PerkinElmer) or HRP-conjugated goat anti-mouse (10148784, 1:5,000, PerkinElmer).

### RNA Interference

siRNAs specific for PKR (SASI_Hs01_00019634), TLR3 (SASI_Hs01_00231802), RIG-I (SASI_Hs02_00345407), MDA5 (SASI_Hs01_00171929), LGP2 (SASI_Hs01_00150553), DHX29 (SASI_Hs02_00352587), TRIF (SASI_Hs01_00226929), and STING (SASI_Hs01_00031030) were purchased from Sigma-Aldrich. MAVS siRNA was from Dachmocon. Transfection of siRNA was conducted using Lipofectamine RNAiMAX (Invitrogen) according to the manufacturer’s instructions. After 48 h, the knockdown level was assessed by qPCR, and the cells were used for subsequent experiments.

### Confocal Microscopy

Cells were cultured and transfected with rhodamine-labeled Poly(dA:dT) for 3 h. Images were captured with an Olympus confocal microscope at the Institute of Immunology, the First Hospital of Jilin University. Image deconvolution was carried out with ImageJ (National Institutes of Health).

### RNA Sequencing

Total RNA was extracted using the EasyPure RNA kit (TransGen, Beijing, China) according to the manufacturer’s instructions. Approximately 1,000 ng of RNA was used for library preparation and subsequent sequencing on an Illumina HiSeq 4000 platform. Reads were aligned to the reference genome (GRCh38.p13) by TopHat2 and HISAT2 software. Differentially expressed genes were analyzed by DEGseq software, and heatmap was generated by GraphPad Prism 7 (GraphPad Software, San Diego, CA, United States).

### Statistical Analysis

Statistical differences were determined by using the two-tailed Student’s *t*-test with GraphPad Prism 7 (GraphPad Software, San Diego, CA, United States); *p*-values less than 0.05 were considered statistically significant.

## Results

### Expression of Major Molecules in the TLR3–TRIF, RLR–MAVS, and cGAS–STING Pathways in Human Epithelial Cancer Cell Lines

**FIGURE 1:**
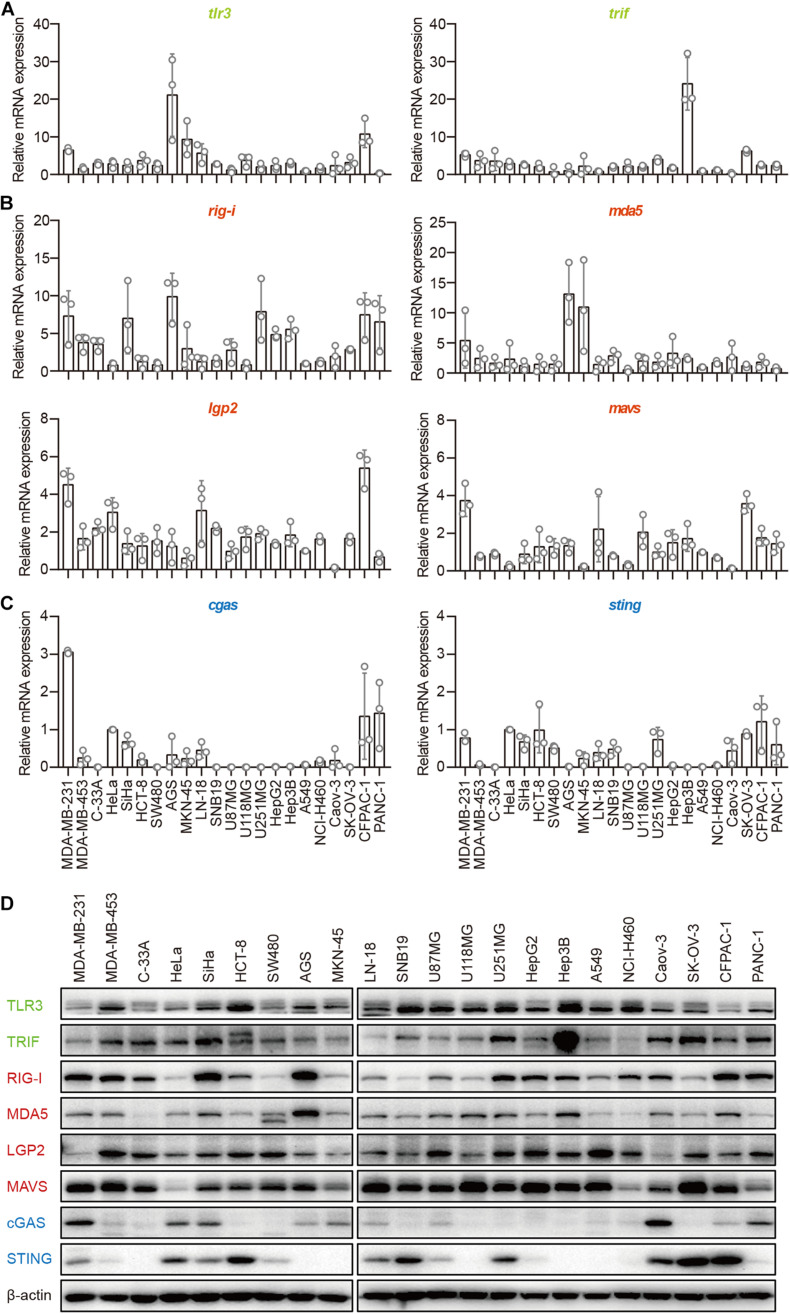
Human epithelial cancer cells express all major molecular nucleic acid sensors. RNA was extracted from 22 human epithelial cancer cell lines. The expression of major molecules in the TLR–TRIF pathway **(A)**, RLR–MAVS pathway **(B)**, and cGAS–STING pathway **(C)** was detected by qPCR. **(D)** Twenty-two human epithelial cancer cell lines were lysed and subjected to western blotting to detect the expression of TLR3, TRIF, RIG-I, MDA5, LGP2, MAVS, cGAS, and STING. β-Actin was the loading control. Data are representative of three independent experiments.

The TLR3–TRIF, RLR–MAVS, and cGAS–STING pathways are the most important signaling pathways in immune cells in the defense against invading pathogens. To investigate their roles in human epithelial cancer cells, we collected 22 human epithelial cancer cell lines derived from nine cancer types, including breast cancer, cervical cancer, colorectal cancer, gastric cancer, glioma, human hepatocellular cancer, lung cancer, human ovarian cancer, and human pancreatic cancer (Supplementary Table 1) and detected the major molecules in the TLR3–TRIF, RLR–MAVS, and cGAS–STING pathways in these cells, including TLR3, TRIF, RIG-I, MDA5, LGP2, MAVS, cGAS, and STING. The results showed that TLR3, TRIF, RIG-I, MDA5, LGP2, and MAVS, which were reported to be expressed in the A549 cell line, were expressed in all these cells lines at both the mRNA and protein levels (Figures 1A,B,D), indicating that the TLR–TRIF and RLR–MAVS pathways are constitutively intact in all cancer cells. Interestingly, cGAS was expressed in 13 of the 22 cell lines, and STING was expressed in 16 of the 22. cGAS and STING were co-expressed in 10 of the 22 (45.5%) cell lines at both the mRNA and protein levels (Figures 1C,D). cGAS and STING expression in HeLa cells served as the positive control.

### Human Epithelial Cancer Cells Preferentially Respond to RNA but Not DNA

Next, we transfected these cell lines with 5′-ppp-dsRNA, Poly(A:U), high-molecular-weight Poly(I:C) [Poly(I:C)-HMW], low-molecular-weight Poly(I:C) (Poly(I:C)-LMW), immunostimulatory dsDNA (ISD), HSV 60-mer dsDNA (HSV60), VACV 70-mer dsDNA (VACV70), Poly(dG:dC), 3′3′-cGAMP, and Poly(dA:dT), which induce IFN-β secretion by immune cells, in the presence of Lipofectamine 2,000. The mechanisms of nucleic acid-induced IFN-β secretion are summarized in Supplementary Table 2. To ascertain the capability of these nucleic acids in the cytosol to induce IFN-β, we transfected the cell lines with rhodamine-labeled Poly(dA:dT) and photographed them using a fluorescence confocal microscope. We found that rhodamine-labeled Poly(dA:dT) could be delivered into the cytosol via Lipofectamine 2,000 in all the cell lines (Supplementary Figures 1A–V). However, there was no rhodamine-labeled Poly(dA:dT) in the cytosol of PANC-1 cells when they were cocultured with rhodamine-labeled Poly(dA:dT) (Supplementary Figure 1W).

**FIGURE 2:**
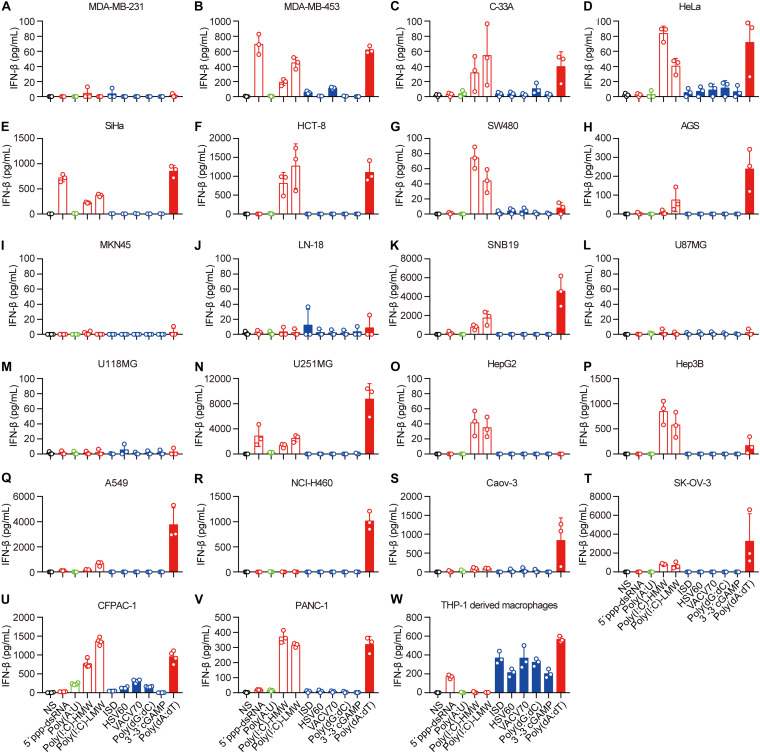
Human epithelial cancer cells preferentially sense RNA but not DNA. **(A)** MDA-MB-231, **(B)** MDA-MB-453, **(C)** C-33A, **(D)** HeLa, **(E)** SiHa, **(F)** HCT-8, **(G)** SW480, **(H)** AGS, **(I)** MKN45, **(J)** LN-18, **(K)** SNB19, **(L)** U87MG, **(M)** U118MG, **(N)** U251MG, **(O)** HepG2, **(P)** Hep3B, **(Q)** Caov-3, **(R)** SK-OV-3, **(S)** A549, **(T)** NCI-H460, **(U)** CFPAC-1, **(V)** PANC-1, and **(W)** THP-1-derived macrophages were transfected using Lipofectamine 2,000 with 5 μg/ml of the following nucleic acids: 5′-ppp-dsRNA, Poly(A:U), high-molecular-weight Poly(I:C) (Poly(I:C)-HMW), low-molecular-weight Poly(I:C) (Poly(I:C)-LMW), immunostimulatory dsDNA (ISD), HSV 60-mer dsDNA (HSV60), VACV 70-mer dsDNA (VACV70), Poly(dG:dC), 3′3′-cGAMP, and Poly(dA:dT). Culture supernatants were harvested 18 h later to detect IFN-β secretion by ELISA. All data are shown as the mean ± SD of at least three independent experiments.

Then, we detected IFN-β secretion induced by cytosolic nucleic acids. IFN-β secretion was markedly elevated in the majority of the cell lines transfected with 5′-ppp-dsRNA, Poly(I:C)-HMW, Poly(I:C)-LMW, or Poly(dA:dT) (Figures 1B–H,K,N–V), indicating that the RLR–MAVS pathway functions in these cell lines. However, several cell lines, including MDA-MB-231, MKN-45, LN-18, U87MG, and U118MG, did not sense any of these cytosolic nucleic acids (Figures 2A,I,J,L,M). There was no response of any cell line to ISD, HSV60, VACV70, Poly(dG:dC), or 3′,3′-cGAMP as indicated by the lack of IFN-β secretion (Figures 2A–V). Meanwhile, THP-1-derived macrophages showed a significant response to ISD, HSV60, VACV70, Poly(dG:dC), 3′,3′-cGAMP, and Poly(dA:dT) (Figure 2W), implying that all these cancer cells are defective in the STING-dependent sensing pathway.

Because of the lack of response to cytosolic ISD, HSV60, VACV70, Poly(dG:dC), or 3′3′-cGAMP in all the cell lines, we transfected PANC-1 cells with different concentrations of 5′-ppp-dsRNA, Poly(A:U), Poly(I:C)-HMW, Poly(I:C)-LMW, ISD, HSV60, VACV70, Poly(dG:dC), 3′3′-cGAMP, and Poly(dA:dT). The results showed that IFN-β was only induced by 5′-ppp-dsRNA, Poly(I:C)-HMW, Poly(I:C)-LMW, and Poly(dA:dT) in a dose-dependent manner (Supplementary Figures 2A,C,D,J), while there was no response to ISD, HSV60, VACV70, Poly(dG:dC), or 3′3′-cGAMP, regardless of the concentration (Supplementary Figures 2B,E–I).

Thus, the RLR–MAVS pathway is functional in most human epithelial cancer cells, but the cGAS–STING pathway is inactive.

### Human Epithelial Cancer Cells Sense Cytosolic RNA via RIG-I, but Not TLR3 or MDA5

**FIGURE 3:**
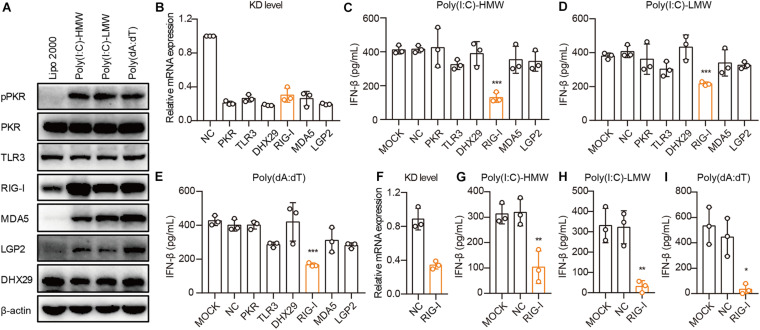
Human epithelial cancer cells sense cytosolic RNA via RIG-I, but not TLR3 or MDA5. **(A)** PANC-1 cells were transfected with Poly(I:C)-HMW, Poly(I:C)-LMW, and Poly(dA:dT) for 9 h. Cells were then lysed and subjected to western blotting to detect the expression of pPKR, PKR, TLR3, RIG-I, MDA5, LGP2, and DHX29, with β-actin as the loading control. Data are representative of three independent experiments. **(B)** PANC-1 cells were transfected using Lipofectamine RNAiMAX with non-silencing negative control siRNA (siNC) or siRNA specific for PKR, TLR3, RIG-I, MDA5, LGP2, and DHX29. Forty-eight hours later, RNA was extracted to detect the knockdown level (KD level) by qPCR. **(C–E)** Silenced PANC-1 cells were transfected with Poly(I:C)-HMW, Poly(I:C)-LMW, and Poly(dA:dT) for 18 h. Culture supernatants were harvested, and IFN-β secretion was measured by ELISA. **(F)** HCT-8 cells were transfected using Lipofectamine RNAiMAX with non-silencing negative control siRNA (siNC) or siRNA specific for RIG-I to detect the knockdown (KD) level by qPCR. **(G–I)** Silenced HCT-8 cells were transfected with Poly(I:C)-HMW, Poly(I:C)-LMW, and Poly(dA:dT) for 18 h. Culture supernatants were harvested, and IFN-β secretion was measured by ELISA. All data are shown as mean ± SD of at least three independent experiments (*p < 0.05, **p < 0.01, ***p < 0.001).

To demonstrate the mechanisms through which cancer cells sense cytosolic Poly(I:C) and Poly(dA:dT), we first examined the activation and expression of PKR, TLR3, RIG-I, MDA5, LGP2, and DHX29 stimulated by Poly(I:C) and Poly(dA:dT) in PANC-1 cells. We found that these receptors were constitutively expressed in PANC-1 cells and that the phosphorylation of PKR and the expression of RIG-I, MDA5, and LGP2 were significantly increased upon Poly(I:C) and Poly(dA:dT) transfection in PANC-1 cells (Figure 3A).

Next, we used small interfering RNA (siRNA) to knock down these receptors in PANC-1 cells. siRNA for each receptor significantly knocked down their expression, as assessed by real-time quantitative PCR (qPCR) (Figure 3B). Upon transfection with Poly(I:C)-HMW, Poly(I:C)-LMW, and Poly(dA:dT), knockdown of RIG-I, but not MDA5, which senses Poly(I:C)-HMW (Kato et al., 2008) or TLR3, resulted in a significant decrease in the secretion of IFN-β in PANC-1 cells (Figures 3C–E). Furthermore, RIG-I knockdown also markedly reduced IFN-β secretion after transfection with Poly(I:C)-HMW, Poly(I:C)-LMW, and Poly(dA:dT) in a colorectal cancer cell line, HCT-8 (Figures 3F–H).

It is reported that Poly(dA:dT) is transcribed into dsRNA by RNA polymerase III, which is then recognized by RIG-I (Ablasser et al., 2009; Chiu et al., 2009). To investigate the role of RNA polymerase III in the RIG-I signaling pathway in human cancer cells, PANC-1 and A549 cells were treated with the RNA polymerase III inhibitor ML-60218, and the expression of IFN-β induced by Poly(dA:dT) was determined. ML-60218 treatment had no effect on the Poly(dA:dT)-induced IFN-β expression in human cancer cells (Supplementary Figures 3A,B) but significantly inhibited this expression in the 293T cells (Supplementary Figure 3C), indicating that RNA polymerase III is not involved in the RIG-I signaling pathway in human cancer cells.

Therefore, RIG-I, rather than TLR3 or MDA5, is required for sensing cytosolic nucleic acids in human cancer cells.

### Cytosolic Poly(I:C) and Poly(dA:dT) Activate the MAVS–IRF3 Pathway in Human Epithelial Cancer Cells

**FIGURE 4:**
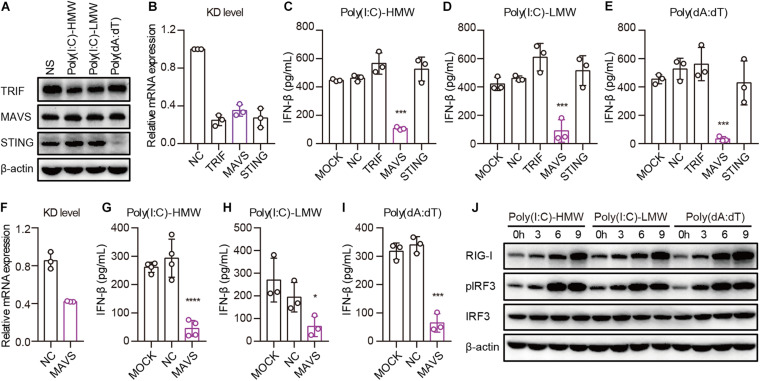
Cytosolic Poly(I:C) and Poly(dA:dT) activate the MAVS–IRF3 pathway in human epithelial cancer cell lines. **(A)** PANC-1 cells were transfected with Poly(I:C)-HMW, Poly(I:C)-LMW, and Poly(dA:dT) for 9 h, after which the cells were lysed and subjected to western blotting to detect the expression of TRIF, MAVS, and STING; β-actin was the loading control. Data are representative of three independent experiments. **(B)** PANC-1 cells were transfected using Lipofectamine RNAiMAX with siNC or siRNA specific for TRIF, MAVS, and STING. Forty-eight hours later, RNA was extracted to detect the KD level by qPCR. **(C–E)** Silenced PANC-1 cells were transfected with Poly(I:C)-HMW, Poly(I:C)-LMW, and Poly(dA:dT) for 18 h. Culture supernatants were harvested, and IFN-β secretion was measured by ELISA. All data are shown as the mean ± SD of at least three independent experiments (*p < 0.05, **p < 0.01, ***p < 0.001). **(F)** HCT-8 cells were transfected using Lipofectamine RNAiMAX with siNC or siRNA specific for MAVS. Forty-eight hours later, RNA was extracted to detect the knockdown (KD) level by qPCR. **(G–I)** Silenced HCT-8 cells were transfected with Poly(I:C)-HMW, Poly(I:C)-LMW, and Poly(dA:dT) for 18 h. Culture supernatants were harvested, and IFN-β secretion was measured by ELISA. All data are shown as mean ± SD of at least three independent experiments (*p < 0.05, **p < 0.01, ***p < 0.001). **(J)** PANC-1 cells were transfected with Poly(I:C)-HMW, Poly(I:C)-LMW, or Poly(dA:dT) for 3, 6, or 9 h. Next, they were lysed and subjected to western blotting to detect the expression of β-actin, RIG-I, and total and phosphorylated IRF3. Data are representative of at least three independent experiments.

Invading pathogens are recognized by PRRs, after which adaptors are recruited for the activation of downstream signaling pathways, leading to the secretion of type I IFN. To determine which adaptor is necessary for RIG-I to activate downstream signaling pathways in cancer cells, we studied the expression of TRIF, MAVS, and STING in PANC-1 cells. TLR3, MAVS, and STING were constitutively expressed and showed no difference after transfection with Poly(I:C)-HMW, Poly(I:C)-LMW, or Poly(dA:dT) (Figure 4A). Then, these molecules were knocked down by siRNA, and the expression of these molecules decreased significantly at the mRNA level (Figure 4B). Upon transfection of PANC-1 cells with cytosolic Poly(I:C)-HMW, Poly(I:C)-LMW, and Poly(dA:dT), IFN-β secretion was markedly decreased after the knockdown of MAVS, but not of TRIF or STING (Figures 4C–E). Similar results were obtained in the human colorectal cancer cell line HCT-8 (Figures 4F–H).

Furthermore, we detected the activation of a downstream pathway involved in IFN-β secretion. We found that IRF-3 was markedly phosphorylated upon transfection of PANC-1 cells with Poly(I:C)-HMW, Poly(I:C)-LMW, and Poly(dA:dT) in a time-dependent manner (Figure 4I).

Taken together, these results indicate that human epithelial cancer cells respond to cytosolic nucleic acids via the RIG-I–MAVS–IRF3 signaling pathway.

### cGAS–STING Pathway Is Activated in cGAS and STING Intact Human Epithelial Cancer Cells

**FIGURE 5:**
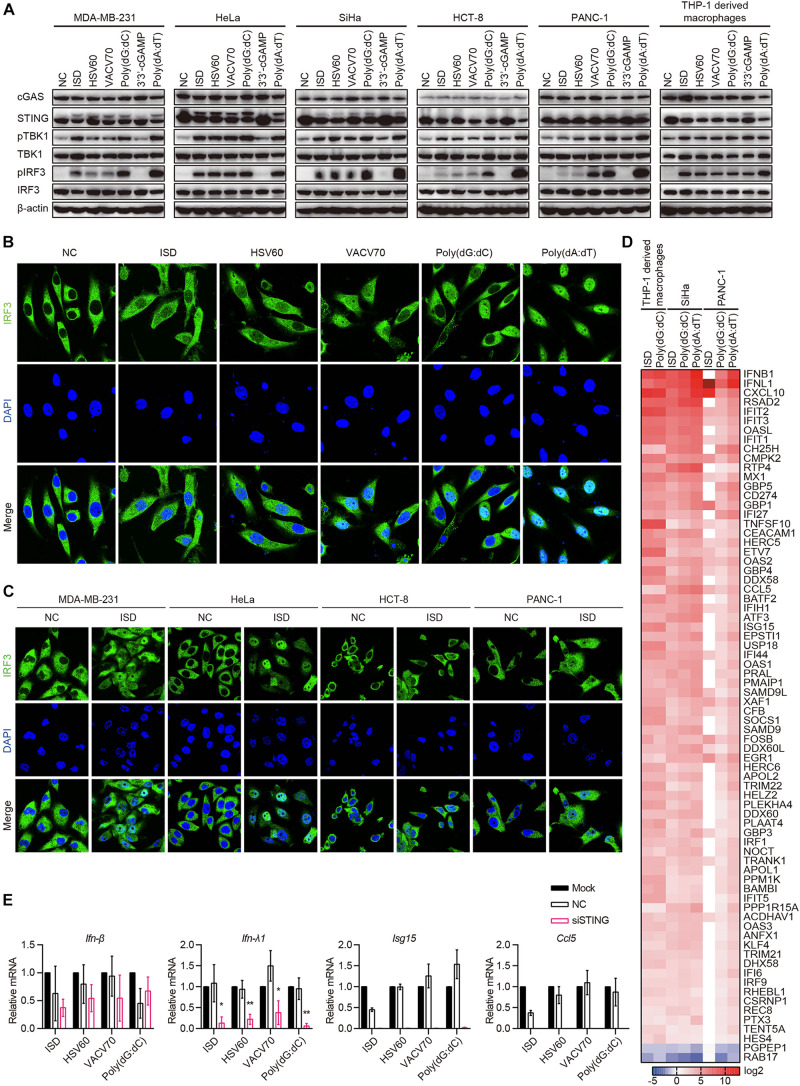
cGAS–STING pathway is activated in cGAS and STING intact human epithelial cancer cells. **(A)** MDA-MB-231, HeLa, SiHa, HCT-8, PANC-1, and THP-1-derived macrophages were transfected with ISD, HSV60, VACV70, Poly(dG:dC), 3′3′-cGAMP, and Poly(dA:dT). Six hours later, cells were lysed and subjected to western blotting to detect the phosphorylation of TBK1 and IRF3 and the expression of cGAS, STING, TBK1, and IRF3. β-Actin was used as the loading control. Data are representative of at least three independent experiments. **(B)** SiHa cells were transfected with ISD, HSV60, VACV70, Poly(dG:dC), 3′3′-cGAMP, and Poly(dA:dT). Six hours later, cells were fixed, and immunofluorescence was performed. Data are representative of at least three independent experiments. **(C)** MDA-MB-231, HeLa, HCT-8, and PANC-1 were transfected with ISD for 6 h, and then cells were fixed to perform immunofluorescence analysis. Data are representative of at least three independent experiments. **(D)** THP-1-derived macrophages were transfected with ISD and Poly(dG:dC), and SiHa and PANC-1 cells were transfected with ISD, Poly(dG:dC), 3′3′-cGAMP, and Poly(dA:dT) for 6 h. Then, RNA was extracted for sequencing. Heatmap was plotted according to differentially expressed genes in different groups. **(E)** SiHa cells were transfected using Lipofectamine RNAiMAX with siNC or siRNA specific for STING for 48 h. Silenced HCT-8 cells were then transfected with ISD, HSV60, VACV70, and Poly(dG:dC). Eighteen hours later, RNA was extracted; and the expression of IFN-β, IFN-λ, ISG15, and CCL5 was detected by qPCR. Data are representative of at least three independent experiments. (*p < 0.05, **p < 0.01, ***p < 0.001).

Because cGAS and STING were co-expressed in 10 of the 22 cell lines, and there was no IFN-β secretion in all cell lines stimulated with ISD, HSV60, VACV70, Poly(dG:dC), and 3′3′-cGAMP, we aimed to determine whether the cGAS–STING pathway was activated by ISD, HSV60, VACV70, Poly(dG:dC), and 3′3′-cGAMP. MDA-MB-231, HeLa, SiHa, HCT-8, and PANC-1 cells, which expressed both cGAS and STING, were transfected with ISD, HSV60, VACV70, Poly(dG:dC), 3′3′-cGAMP, and Poly(dA:dT). As THP-1-derived macrophages, STING was phosphorylated or degraded after transfection with ISD, HSV60, VACV70, Poly(dG:dC), and Poly(dA:dT) (Figure 5A). TBK1 and IRF3 were also phosphorylated in these cell lines after transfection, although their phosphorylation was weak in HCT-8 cells stimulated with ISD, HSV60, and VACV70 as well as in PANC-1 cells stimulated with ISD and HSV60 (Figure 5A). Interestingly, STING, TBK1, and IRF3 were not phosphorylated after transfection with 3′3′-cGAMP in these cancer cell lines (Figure 5A).

Upon stimulation, IRF3 is phosphorylated and translocated to the nucleus in immune cells. Thus, we transfected SiHa cells with ISD, HSV60, VACV70, Poly(dG:dC), and Poly(dA:dT) and confirmed whether IRF3 was translocated to the nucleus in these cells. We found that IRF3 was significantly translocated to the nucleus after transfection with ISD, HSV60, VACV70, and Poly(dG:dC), although the translocation was weak compared with that with Poly(dA:dT) (Figure 5B). The same results were obtained in MDA-MB-231, HeLa, HCT-8, and PANC-1 cell lines (Figure 5C).

Next, we transfected SiHa and PANC-1 cells with ISD, Poly(dG:dC), and Poly(dA:dT) and performed RNA sequencing. The results showed that the expression profiles in SiHa cells stimulated with ISD, Poly(dG:dC), and Poly(dA:dT) as well as in PANC-1 cells stimulated with Poly(dG:dC) and Poly(dA:dT) were the same as those in THP-1-derived macrophages stimulated with ISD and Poly(dG:dC) (Figure 5D). Expression profiles in PANC-1 cells stimulated with ISD differed from those in other cells because these cells did not respond to ISD, as demonstrated by the lack of TBK1 and IRF3 phosphorylation (Figure 5D). Interestingly, STING knockdown in SiHa cells suppressed the expression of IFN-λ1, ISG15, and CCL5 induced by ISD, HSV60, VACV70, and Poly(dG:dC); however, the expression of IFN-β was not decreased (Figures 5E,F). These data suggest that cGAS and STING intact human epithelial cancer cells sense cytosolic DNA through the cGAS–STING signaling pathway to produce cytokines, chemokines, and ISGs. Furthermore, novel pathways that control the secretion of IFN-β may exist.

## Discussion

We report that TLR3, TRIF, RIG-I, MDA5, LGP2, and MAVS were expressed in/on all 22 human epithelial cancer cell lines studied, but only cytosolic 5′-ppp-dsRNA, Poly(I:C)-HMW, Poly(I:C)-LMW, and/or Poly(dA:dT) could induce IFN-β secretion via the RIG-I–MAVS–IRF3 signaling pathway in most of the cell lines. Although both cGAS and STING were co-expressed in 10 of the 22 cell lines, none of these cell lines secreted IFN-β induced by cytosolic ISD, HSV60, VACV70, Poly(dG:dC), and 3′3′-cGAMP, irrespective of the expression pattern of cGAS and STING. Further experiments revealed that the cGAS–STING pathway was activated, as revealed by TBK1 and IRF3 phosphorylation and IFN-β and ISGs mRNA expression induced by cytosolic ISD, HSV60, VACV70, and Poly(dG:dC) in cGAS and STING intact cell lines. Therefore, most human epithelial cancer cell lines respond to cytosolic RNA through the RIG-I–MAVS–IRF3 signaling pathway, while cytosolic DNA was sensed by the cGAS–STING signaling pathway in cGAS and STING intact human epithelial cancer cell lines (Supplementary Figure 4).

RIG-I-like receptors play important roles in recognizing cytosolic RNA molecules. They have been found to be expressed in several human cancer cells and tissues (hepatocytes, intestinal epithelial cells, lung epithelial cells, primary human astrocytes, and glioblastoma), and these recognize cytosolic Poly(I:C) and viruses (Li et al., 2005; Hirata et al., 2007; Wang et al., 2009; Furr et al., 2010; Broquet et al., 2011; Glas et al., 2013; Sugimoto et al., 2014). We found that RIG-I, MDA5, LGP2, and MAVS were expressed in all human epithelial cancer cell lines analyzed in the current study. Results of functional analyses revealed that most of cancer cells responded to cytosolic Poly(I:C). This finding was consistent with that of previous studies. Additionally, cytosolic 5′-ppp-dsRNA and Poly(dA:dT) were found to activate most of the cell lines, as indicated by IRF3 phosphorylation and IFN-β secretion. Knockdown experiments showed that IFN-β secretion was inhibited by RIG-I and MAVS knockdown, demonstrating that most of cancer cells responded to cytosolic RNA through the RIG-I–MAVS–IRF3 signaling pathway. Moreover, we found that several cell lines (MDA-MB-231, MKN-45, LN-18, U87MG, and U118MG) sensed none of these cytosolic nucleic acids, indicating that hitherto unknown sensors might exist in these cell lines.

Several experimental studies have reported the expression of cGAS and/or STING in human melanoma, colorectal cancer, Merkel cell carcinoma, and ovarian cancer (Xia et al., 2016a,b; de Queiroz et al., 2019; Liu et al., 2020). However, their expression in other human cancer cells remains unknown. In the current study, we found that cGAS and STING were each expressed in approximately 59 and 73% of the cell lines examined, respectively, and cGAS and STING were co-expressed in only 45.5% of these cell lines. Although one study reported that cGAS and STING were highly expressed in various cancer tissues using bioinformatics analysis (An et al., 2019), their expression remains to be confirmed in more cell lines.

Previous studies and our present study reported that human epithelial cancer cell lines cannot respond to cytosolic DNA regardless of the expression pattern of cGAS and STING. Reportedly, lung adenocarcinoma-intrinsic glycogen branching enzyme (GBE1) antagonizes the expression and activation of STING (Li et al., 2019). SOX2 promotes the degradation of STING protein in an autophagy-dependent manner in neck squamous cell carcinoma (Tan et al., 2018). Loss of MUS81 leads to the attenuation of STING-dependent type I interferon expression in prostate cancer cells (Ho et al., 2016). HER2 is strongly associated with STING and recruits AKT1 to directly phosphorylate TBK1 (Wu et al., 2019). Colon cancer cells hijack caspase-9 signaling to suppress the radiation-induced mitochondrial DNA–cGAS–STING sensing pathway and limit the secretion of type I IFNs (Han et al., 2020). However, in the present study, we found that cytosolic ISD, HSV60, VACV70, and Poly(dG:dC) induced the phosphorylation of TBK1 and IRF3, and the expression profiles in cGAS and STING intact human epithelial cancer cells induced by cytosolic DNA were the same as those in THP-1-derived macrophages. Further experiments revealed that the expression of IFN-λ1, ISG15, and CCL5 induced by cytosolic ISD, HSV60, VACV70, and Poly(dG:dC) was impaired by the knockdown of STING, while the expression of IFN-β was STING-independent. Thus, cGAS and STING intact human epithelial cancer cells can sense cytosolic DNA through the cGAS–STING signaling pathway, and there may be novel pathways and molecules to control the production and secretion of IFN-β.

In conclusion, we ascertained that most human cancer cells respond to cytosolic RNA through the RIG-I–MAVS–IRF3 signaling pathway, while the cGAS–STING pathway is activated despite the absence of IFN-β secretion in cGAS and STING intact human cancer cell lines. Our present findings are relevant in developing strategies for targeting nucleic acid receptors in cancer immunotherapy.

## Data Availability Statement

The raw data analyzed during the current study are available from the corresponding author upon reasonable request.

## Author Contributions

Y-JL and JC designed and supervised the project. YQ designed and conducted the experiments. S-SZ, BG, and GZ acquired the data. YQ, YJ, and SZ analyzed the data and prepared figures. SD and GZ cultured cell lines. YQ, Y-JL, and JC wrote and revised the manuscript. All authors contributed to the article and approved the submitted version.

## Conflict of Interest

The authors declare that the research was conducted in the absence of any commercial or financial relationships that could be construed as9 a potential conflict of interest.
