# Supplementary material for: Human Cancer Cells Sense Cytosolic Nucleic Acids Through the RIG-I–MAVS Pathway and cGAS–STING Pathway
Source: Front Cell Dev Biol. 2021 Jan 8;8:606001. doi: 10.3389/fcell.2020.606001 (PMC7820189; doi:10.3389/fcell.2020.606001)
Supplement: Supplementary file 1 [file Data_Sheet_1.PDF]

Supplementary materials:

Table S1. Human epithelial cancer cell lines used in this study

**Type**	**Cell line**	**Description**
Breast cancer	MDA-MB-231	Her2 (-)
	MDA-MB-453	Her2 (+)
Cervical cancer	C-33A	Cervical adenocarcinoma (No infection)
	Hela	Cervical adenocarcinoma (HPV-18 infection)
	SiHa	Cervical adenocarcinoma (HPV-16 infection)
Colorectal cancer	HCT-8	Ileocecal colorectal adenocarcinoma
	SW480	Dukes' type B, colorectal adenocarcinoma
Gastric cancer	AGS	Gastric carcinoma
	MKN45	Gastric carcinoma, poorly differentiated
Glioma	LN-18	Glioblastoma; glioma(IV)
	SNB19	Astrocytoma cells
	U118MG	Glioblastoma; astrocytoma (IV)
	U251	Astrocytoma cells
	U87MG	Glioblastoma; astrocytoma (IV)
Hepatocellular cancer	HepG2	Hepatocellular carcinoma (No infection)
	Hep3B	Hepatocellular carcinoma (HBV infection)
Lung cancer	A549	Lung carcinoma
	NCI-H460	Large cell lung cancer
Ovarian cancer	Caov-3	adenocarcinoma (ovary)
	SK-OV-3	adenocarcinoma (ovary: ascites)
Pancreatic cancer	CFPAC-1	Ductal adenocarcinoma
	PNAC-1	Pancreatic carcinoma

Table S2. Nucleic acids used in this study

Name	Type	Description
5'ppp-dsRNA	dsRNA	a synthetic ligand for RIG-I [1].
Poly(A:U)	A synthetic double stranded RNA	signals only through TLR3 [2].
Poly(I:C)	A synthetic double stranded RNA	recognized by TLR3 and cytosolic RNA helicases RIG-I and MDA-5 [3, 4].
ISD Naked	Non-CpG oligomer from the *Listeria monocytogenes* genome.	mediated by the STING-TBK1-IRF3 signaling axis [5, 6].
HSV-60 Naked	A 60 bp oligonucleotide containing viral DNA motifs	induces IFN-β in a TLR-independent, but STING-, TBK1- and IRF3-dependent manner. HSV-60 is recognized by DDX41 and IFI16 [7, 8].
VACV-70 Naked	A 70 bp oligonucleotides containing viral DNA motifs	induces IFN-β in a TLR-independent, but STING-, TBK1- and IRF3-dependent manner. VACV-70 is recognized by DDX41 and IFI16 [7, 8].
Poly(dG:dC)	A synthetic analog of Z-DNA	recognized by cGAS , DAI , DDX41 , IFI16, LRRFIP1, and AIM2 [7-12].
3’3’-cGAMP	A cyclic di-nucleotide	binds STING and subsequently induces TBK1-IRF3-dependent production of IFN-β [13].
Poly(dA:dT)	A synthetic analog of B-DNA	recognized by several sensors, including cGAS, DAI, DDX41, IFI16 and LRRFIP1, AIM2 and RIG-I [7-12, 14-16].

Supplementary references:

1. Wang, Y., Ludwig, J., Schuberth, C., Goldeck, M., Schlee, M., Li, H., et al.(2010). Structural and functional insights into 5'-ppp RNA pattern recognition by the innate immune receptor RIG-I. *Nat Struct Mol Biol*. 17, 781-7. 10.1038/nsmb.1863.
2. de Bouteiller, O., Merck, E., Hasan, U.A., Hubac, S., Benguigui, B., Trinchieri, G., et al.(2005). Recognition of double-stranded RNA by human toll-like receptor 3 and downstream receptor signaling requires multimerization and an acidic pH. *J Biol Chem*. 280, 38133-45. 10.1074/jbc.M507163200.
3. Alexopoulou, L., Holt, A.C., Medzhitov, R., and Flavell, R.A.(2001). Recognition of double-stranded RNA and activation of NF-kappaB by Toll-like receptor 3. *Nature*. 413, 732-8. 10.1038/35099560.
4. Li, K., Chen, Z., Kato, N., Gale, M., Jr., and Lemon, S.M.(2005). Distinct poly(I-C) and virus-activated signaling pathways leading to interferon-beta production in hepatocytes. *J Biol Chem*. 280, 16739-47. 10.1074/jbc.M414139200.
5. Stetson, D.B. and Medzhitov, R.(2006). Recognition of cytosolic DNA activates an IRF3-dependent innate immune response. *Immunity*. 24, 93-103. 10.1016/j.immuni.2005.12.003.
6. Ishikawa, H., Ma, Z., and Barber, G.N.(2009). STING regulates intracellular DNA-mediated, type I interferon-dependent innate immunity. *Nature*. 461, 788-92. 10.1038/nature08476.
7. Unterholzner, L., Keating, S.E., Baran, M., Horan, K.A., Jensen, S.B., Sharma, S., et al.(2010). IFI16 is an innate immune sensor for intracellular DNA. *Nat Immunol*. 11, 997-1004. 10.1038/ni.1932.
8. Zhang, Z., Yuan, B., Bao, M., Lu, N., Kim, T., and Liu, Y.J.(2011). The helicase DDX41 senses intracellular DNA mediated by the adaptor STING in dendritic cells. *Nat Immunol*. 12, 959-65. 10.1038/ni.2091.
9. Yang, P., An, H., Liu, X., Wen, M., Zheng, Y., Rui, Y., et al.(2010). The cytosolic nucleic acid sensor LRRFIP1 mediates the production of type I interferon via a beta-catenin-dependent pathway. *Nat Immunol*. 11, 487-94. 10.1038/ni.1876.
10. Takaoka, A., Wang, Z., Choi, M.K., Yanai, H., Negishi, H., Ban, T., et al.(2007). DAI (DLM-1/ZBP1) is a cytosolic DNA sensor and an activator of innate immune response. *Nature*. 448, 501-5. 10.1038/nature06013.
11. Wu, J., Sun, L., Chen, X., Du, F., Shi, H., Chen, C., et al.(2013). Cyclic GMP-AMP is an endogenous second messenger in innate immune signaling by cytosolic DNA. *Science*. 339, 826-30. 10.1126/science.1229963.
12. Jones, J.W., Kayagaki, N., Broz, P., Henry, T., Newton, K., O'Rourke, K., et al.(2010). Absent in melanoma 2 is required for innate immune recognition of Francisella tularensis. *Proc Natl Acad Sci U S A*. 107, 9771-6. 10.1073/pnas.1003738107.
13. Zhang, X., Shi, H., Wu, J., Zhang, X., Sun, L., Chen, C., et al.(2013). Cyclic GMP-AMP containing mixed phosphodiester linkages is an endogenous

high-affinity ligand for STING. *Mol Cell*. 51, 226-35. 10.1016/j.molcel.2013.05.022.

14. Chiu, Y.H., Macmillan, J.B., and Chen, Z.J.(2009). RNA polymerase III detects cytosolic DNA and induces type I interferons through the RIG-I pathway. *Cell*. 138, 576-91. 10.1016/j.cell.2009.06.015.
15. Ablasser, A., Bauernfeind, F., Hartmann, G., Latz, E., Fitzgerald, K.A., and Hornung, V.(2009). RIG-I-dependent sensing of poly(dA:dT) through the induction of an RNA polymerase III-transcribed RNA intermediate. *Nat Immunol*. 10, 1065-72. 10.1038/ni.1779.
16. Schlee, M., Roth, A., Hornung, V., Hagmann, C.A., Wimmenauer, V., Barchet, W., et al.(2009). Recognition of 5' triphosphate by RIG-I helicase requires short blunt double-stranded RNA as contained in panhandle of negative-strand virus. *Immunity*. 31, 25-34. 10.1016/j.immuni.2009.05.008.

Supplementary figure legends:

Figure S1. Nucleic acids transfected into human epithelial cancer cells

(A) MDA-MB-231, (B) MDA-MB-453, (C) C-33A, (D) HeLa, (E) SiHa, (F) HCT-8, (G) SW480, (H) AGS, (I) MKN45, (J) LN-18, (K) SNB19, (L) U87MG, (M) U118MG, (N) U251MG, (O) HepG2, (P) Hep3B, (Q) Caov-3, (R) SK-OV-3, (S) A549, (T) NCI-H460, (U) CFPAC-1, and (V) PANC-1 cells were transfected with 0.1 μg/ml rhodamine-labeled poly(dA:dT) for 3 h, then imaged by fluorescence confocal microscope. (W) PANC-1 cells were cocultured with 0.1 μg/ml rhodamine-labeled poly(dA:dT) for 3 h, then imaged by fluorescence confocal microscope as a negative control. Data are representative of at least three independent fields.

Figure S2. IFN-β is induced in dose-dependent manner

(A) 5′ppp-dsRNA, (B) poly(A:U), (C) poly(I:C)-HMW, (D) poly(I:C)-LMW, (E) ISD, (F) HSV60, (G) VACV-70, (H) poly(dG:dC), (I) 3′-3′cGAMP, and (J) poly(dA:dT) were transfected into PANC-1 cells at concentrations of 0.625, 1.25, 2.5, 5, 10, and 20 μg/ml. Eighteen hours later, cell supernatants were harvested, and secretion of IFN-β was detected by ELISA. Data are shown as mean ± SD of at least three independent experiments (* $p < 0.05$, ** $p < 0.01$).

Figure S3. RNA polymerase III is not involved in the RIG-I signaling pathway in human epithelial cancer cells

293T (A), PANC-1 (B), and A549 (C) cells were treated with DMSO or ML-60218 for 12 h and then transfected with poly(dA:dT). After 18 h, total RNA was extracted, and the expression of IFN-β determined.

Figure S4. Schema for stimuli of PRRs and their signaling pathways

The TLR3-TRIF, RIG-I-MAVS, and cGAS-STING pathways are activated by their ligands. Adaptors are then recruited, and TBK1 is phosphorylated. IRF3 is activated by phosphorylated TBK1. pIRF3 is translocated into the nucleus and binds to the IFN-β promoter.
