# Supplementary figures and images for: Human Cancer Cells Sense Cytosolic Nucleic Acids Through the RIG-I–MAVS Pathway and cGAS–STING Pathway
Source: Front Cell Dev Biol. 2021 Jan 8;8:606001. doi: 10.3389/fcell.2020.606001 (PMC7820189; doi:10.3389/fcell.2020.606001)

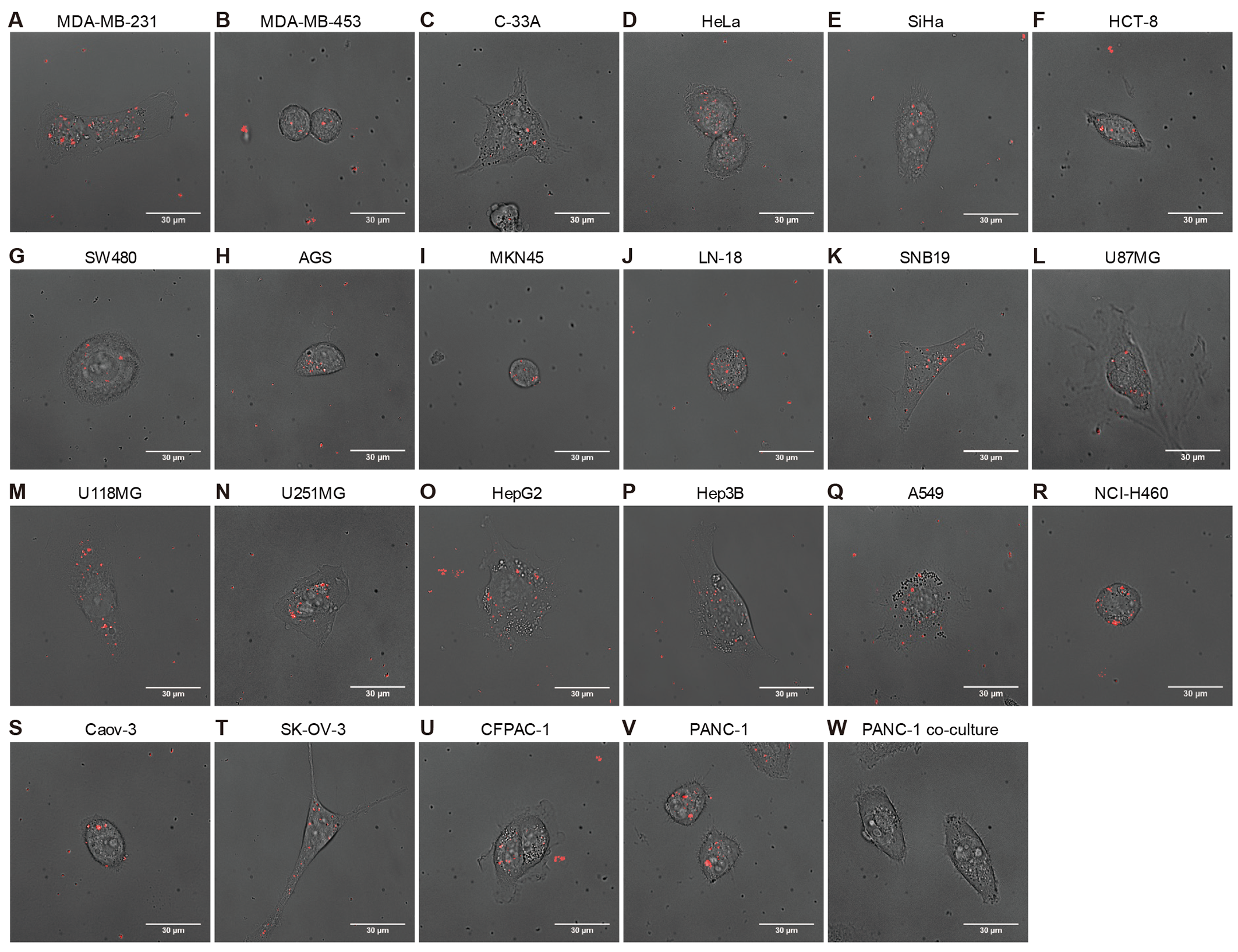

Supplement: Supplementary file 2 [file Image_1.TIF]

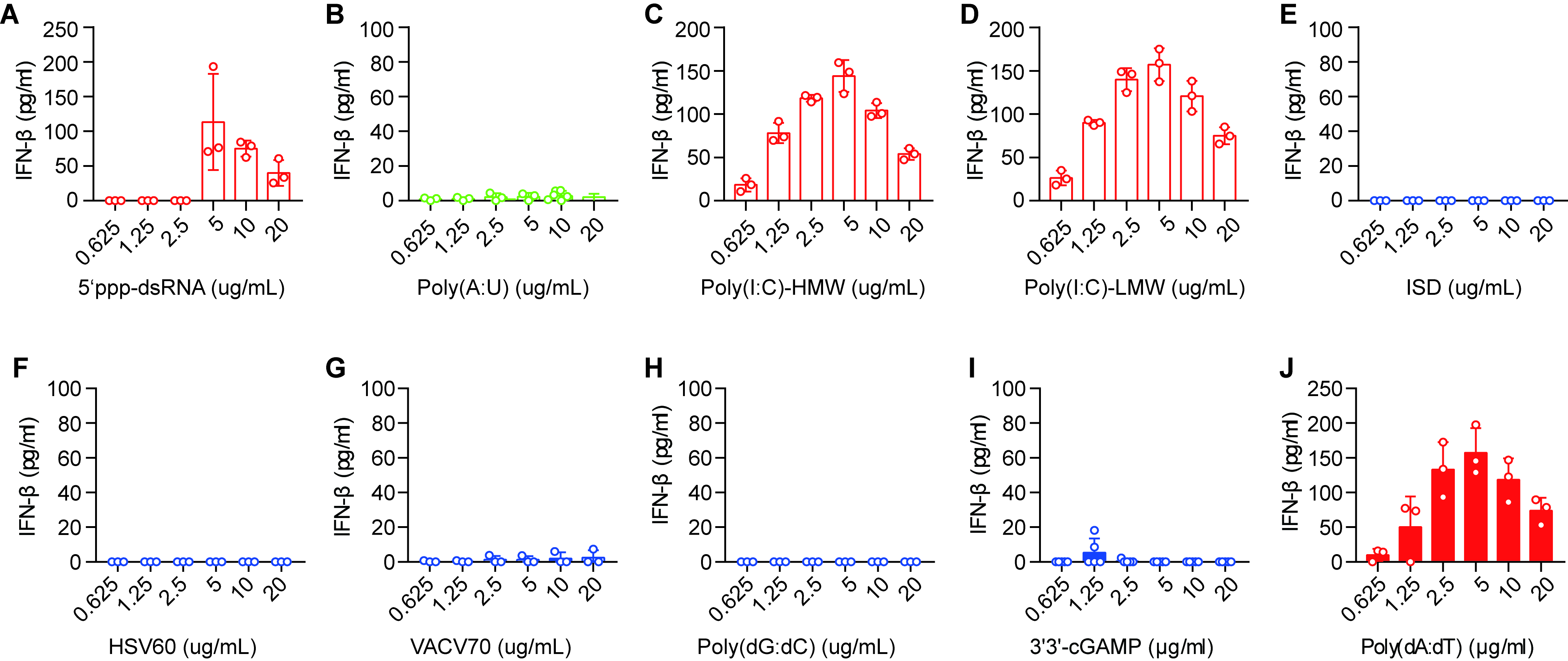

Supplement: Supplementary file 3 [file Image_2.TIF]

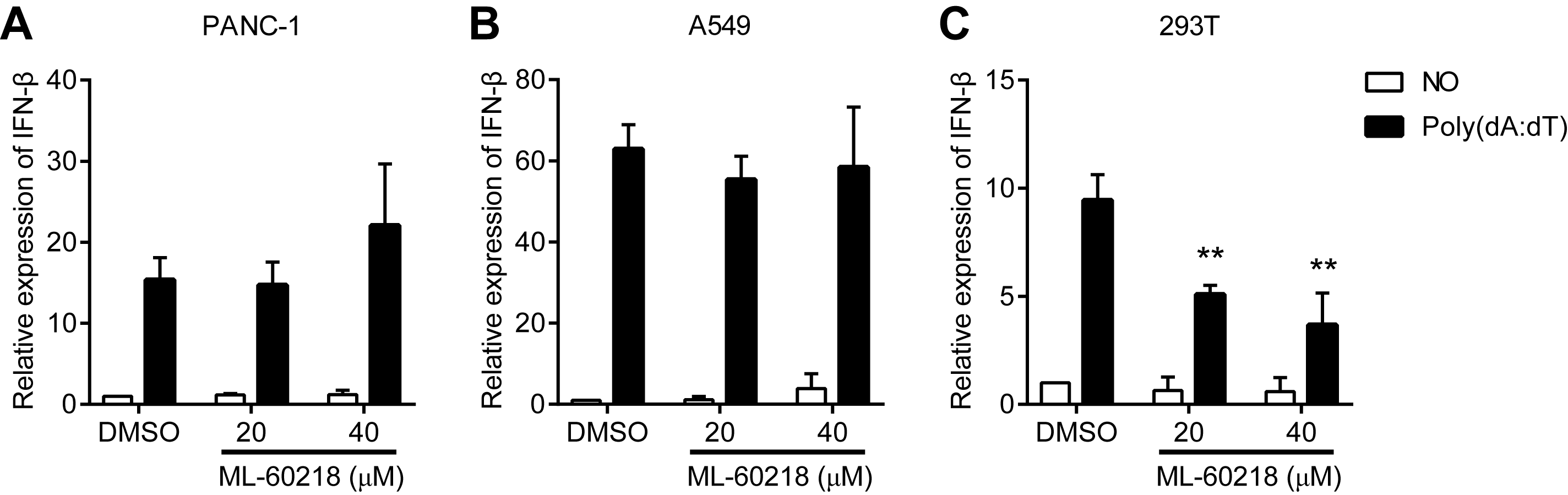

Supplement: Supplementary file 4 [file Image_3.TIF]

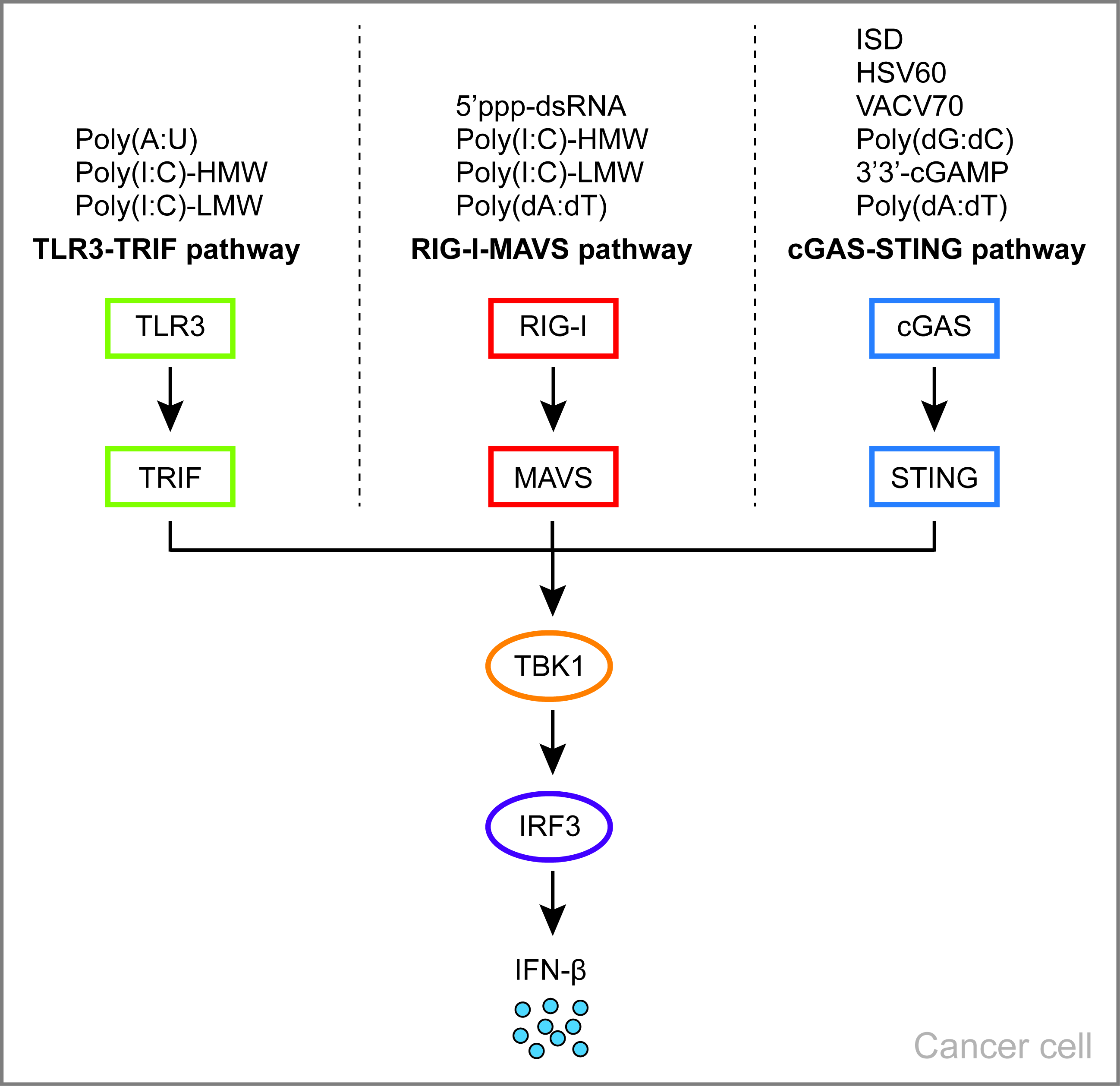

Supplement: Supplementary file 5 [file Image_4.TIF]
